# Evaluating model reduction under parameter uncertainty

**DOI:** 10.1186/s12918-018-0602-x

**Published:** 2018-07-27

**Authors:** Håvard G. Frøysa, Shirin Fallahi, Nello Blaser

**Affiliations:** Department of Mathematics, University of Bergen, Mailbox 7803, Bergen, 5020 Norway

**Keywords:** Model reduction, Parameter uncertainty, Clustering, Systems biology

## Abstract

**Background:**

The dynamics of biochemical networks can be modelled by systems of ordinary differential equations. However, these networks are typically large and contain many parameters. Therefore model reduction procedures, such as lumping, sensitivity analysis and time-scale separation, are used to simplify models. Although there are many different model reduction procedures, the evaluation of reduced models is difficult and depends on the parameter values of the full model. There is a lack of a criteria for evaluating reduced models when the model parameters are uncertain.

**Results:**

We developed a method to compare reduced models and select the model that results in similar dynamics and uncertainty as the original model. We simulated different parameter sets from the assumed parameter distributions. Then, we compared all reduced models for all parameter sets using cluster analysis. The clusters revealed which of the reduced models that were similar to the original model in dynamics and variability. This allowed us to select the smallest reduced model that best approximated the full model. Through examples we showed that when parameter uncertainty was large, the model should be reduced further and when parameter uncertainty was small, models should not be reduced much.

**Conclusions:**

A method to compare different models under parameter uncertainty is developed. It can be applied to any model reduction method. We also showed that the amount of parameter uncertainty influences the choice of reduced models.

**Electronic supplementary material:**

The online version of this article (10.1186/s12918-018-0602-x) contains supplementary material, which is available to authorized users.

## Background

### Modelling of biochemical networks

Biochemical networks consist of chemical reactions between compounds, such as enzymes and metabolites. Through these reactions, the various compounds are consumed and produced. Each of these reactions has a reaction rate (flux) that typically depends on the compound concentrations, giving a dynamical behaviour of the system. The compound concentrations can thus be modelled by systems of ordinary differential equations (ODEs) and such dynamical models of biochemical networks may give biological insight that could not be obtained by modelling the compounds individually. However, the network dynamics may be complex and difficult to model accurately. The chemical reactions could possess advanced kinetics such as activation and inhibition. In addition, the dimensions of the network may be large, for example the central energy metabolism in E. *coli* consists of more than 50 metabolites and 100 reactions [1].

### Model reduction

The potential high complexity of the ODEs in the model represents a major challenge in analysing the dynamics of the system. Model reduction is a method for studying biochemical networks as it aims to identify the main components governing the dynamics of the system. The reduced model should be simpler to analyse, but retain the dynamical behaviour of the original model. There are different approaches to reduce the complexity of biochemical reaction networks, with the most common ones being lumping, sensitivity analysis and time-scale analysis [2–4]. Lumping combines compounds with similar behaviour into pseudo-compounds and considers differential equations involving these lumped pseudo-compounds [5, 6]. By performing parameter sensitivity analysis, the parameters with the least effect on the system output are neglected [7, 8]. In time-scale separation, biological processes are split into fast and slow processes and then the focus is put on the relevant time scale [9–15].

Another challenge in the analysis of complex networks is the lack of information on the kinetic properties of the reactions and parameter values. Reduction approaches that are not influenced by parameter uncertainty or incompleteness are called parameter independent reduction methods. For example, some reduction techniques based on exact lumping methods [5, 6] or qualitative reduction methods [16, 17] are parameter independent. Such reduction methods have been used extensively for signalling networks. For most reduction techniques, including methods based on time-scale separation or sensitivity analysis, the full parametrization of the model is required. In parameter dependent reduction, model parameters can play a significant role in selecting the elements for reduction. For some biochemical networks, the accuracy and validity of the reduced model can be influenced by changing the range of parameters so that the reduced model is only valid locally [3]. For reaction networks with well separated parameter values, reduced models capture the dynamical behaviour of the original model with an acceptable level of accuracy for an extensive range of parameter values [11, 18]. This, however, is not the case for general networks.

While there is a large literature on model reduction techniques, there is a lack of methods for evaluating model reductions. Some ad-hoc methods are the difference or scaled difference between the full and reduced model [5, 9], an error integral [14] and a criterion based on the initial values [10]. We are not aware of any criteria for evaluation of model reductions that takes parameter uncertainty into account. We present a new way to evaluate model reductions that takes parameter uncertainty into account and show the benefit of this method on two example networks.

## Methods

### Mathematical framework

The state variables of the dynamical model are the concentrations of the compounds. These compounds occur in different combinations on the left and right hand side of the chemical reactions of the network, where such a combination is called a complex [14]. For example, the chemical reaction *X*_1_+*X*_2_→*X*_3_ consists of the compounds *X*_1_, *X*_2_ and *X*_3_, and the complexes *X*_1_+*X*_2_ and *X*_3_. The complex on the left hand side of an equation being consumed is called the substrate complex of the reaction and the complex on the right hand side of the reaction being produced is called the product complex. All this information can be represented mathematically by a stoichiometric matrix [1] which gives the structure of the network.

In the notation of Rao et al. [14] the complexes are given by a matrix *Z* where the columns are the non-negative integer stoichiometric coefficients of the different complexes. The internal reactions are given by the linkage matrix *B* where each column corresponds to a reaction. This column is zero except in the rows corresponding to the substrate and product complex where it is -1 and 1, respectively. Let *x*_*i*_(*t*) be the concentration of compound *i* at time *t* and **x**(*t*) the corresponding vector quantity. The dynamics of any biochemical network is given by the system

1 $$ \dot{\mathbf{x}}=ZB\mathbf{v}+Z\mathbf{v}_{b}  $$

of ODEs where *Z* and *B* give the network structure as described above. The vector **v** provides the internal fluxes of the network and **v**_*b*_ the boundary fluxes, i.e. the fluxes entering or leaving the network. As the fluxes typically are functions of **x**, we restrict the internal fluxes **v** to the form

2 $$ v_{j}(\mathbf{x})=k_{j}d_{j}(\mathbf{x})\exp\left(Z_{\mathcal{S}_{j}}^{T}\text{Ln}(\mathbf{x})\right)  $$

considered in [14] where *k*_*j*_ is a kinetic proportionality constant of reaction *j*, *d*_*j*_(**x**) is any function of **x**, $Z_{\mathcal {S}_{j}}$ is the column of *Z* corresponding to the substrate complex of reaction *j* and Ln(**x**) is the mapping defined by (Ln(**x**))_*i*_= ln(*x*_*i*_). Further, let $Z_{\mathcal {S}}$ be the matrix where column *j* is $Z_{\mathcal {S}_{j}}$, i.e. the substrate complex of the reaction.

The dynamical model (1) now has the parameters *k*_*j*_ in addition to potential parameters in **v**_*b*_(**x**) and the functions *d*_*j*_(**x**). A given set of values for such a parametrization will be called a parameter set. The unreduced model described by (1) will be referred to as the full or original model.

### Reduction

We use the reduction procedure of Rao et al. [14] to reduce the model for a given parameter set. The first step in this procedure is to specify a set $\mathcal {M}_{\mathrm {I}}$ of compounds considered to be important in the view of experimental design, e.g. the ones that are possible to measure. Note that the choice of $\mathcal {M}_{\mathrm {I}}$ is subjective, but plays a major role in the reduction as the dynamics of the compounds in $\mathcal {M}_{\mathrm {I}}$ are the ones used to compare the different reduced models. Then, the complexes of the network are divided into two categories. The first category is the complexes containing at least one of the compounds in $\mathcal {M}_{\mathrm {I}}$. These complexes will not be considered for reduction. The other category is the complexes not containing any of the compounds in $\mathcal {M}_{\mathrm {I}}$, and these will be the complexes considered for reduction. The reduction is then based on the assumption that the model approaches some steady state that can be found by integrating the system for a long enough time and that the model is asymptotically stable around the steady state. A complex is reduced by setting its concentration constant equal to the corresponding steady state value of the full model. This can be done simultaneously for any number of complexes.

Having the possibility to reduce any given set of complexes, an iterative method to choose the complexes to be reduced is presented in Rao et al. [14]. It is a greedy method that reduces one complex at the time, always choosing the one yielding the smallest error as defined below. Finally, it stops when an error threshold is reached.

However, since the reduced models are independent of the order of reduction, we consider all possible simultaneous reductions of complexes. Assume now that there are *c* complexes eligible for reduction. It is then possible to reduce anywhere from 0 to *c* complexes, where reducing 0 gives the full model. In total there are 2^*c*^ possible reduced models for a given original model and parameter set. For each of these models, the concentrations of the compounds in $\mathcal {M}_{\mathrm {I}}$ are then used to compare the models. When having *n* different parameter sets for the same original model, we perform the described reduction procedure for all the parameter sets. This yields 2^*c*^ possible reduced models for each parameter set and a total of *n*·2^*c*^ different reduced models.

### Comparing models

We need to be able to compare the dynamics of the different reduced models. In Rao et al. [14] the difference between the original model and a given reduced model is measured by an error integral. Let the concentration at time *t* of compound number *i* be *x*_*ir*_(*t*) and *x*_*if*_(*t*) for the reduced and the full model, respectively. Further, let **x**_*r*_ and **x**_*f*_ be the corresponding vector quantities for all the compounds. Finally, let $n\left (\mathcal {M}_{\mathrm {I}}\right)$ be the number of compounds in $\mathcal {M}_{\mathrm {I}}$ and [0,*T*] the time interval that we evaluate the dynamics over. The error integral is then given by

3 $$ I_{T}\left(\mathbf{x}_{r},\mathbf{x}_{f}\right) = \sum_{i\in\mathcal{M}_{\mathrm{I}}}\frac{1}{Tn\left(\mathcal{M}_{\mathrm{I}}\right)}\int_{0}^{T}\left|1-\frac{x_{ir}(t)}{x_{if}(t)}\right|dt  $$

which gives the average relative difference between the full and reduced model for all the compounds in $\mathcal {M}_{\mathrm {I}}$ over the given time interval. Note that the error integral is non-symmetric in its arguments. However, we need to compare any two (reduced) models without favouring one of them. For this reason we introduce the symmetric error measure

4 $$ E_{T}\left(\mathbf{x}_{1},\mathbf{x}_{2}\right) = \frac{1}{2}\left(I_{T}\left(\mathbf{x}_{1},\mathbf{x}_{2}\right)+I_{T}\left(\mathbf{x}_{2},\mathbf{x}_{1}\right)\right)  $$

where **x**_1_ and **x**_2_ are the compound concentrations of any two (reduced) models. Note that this error measure can be calculated also for two models having different parameters as long as they have the same set $\mathcal {M}_{\mathrm {I}}$.

### Clustering

We use single linkage clustering [19] with the symmetric error as dissimilarity measure to cluster all the *n*·2^*c*^ models with different parameter sets and reductions. Single linkage clustering is an agglomerative clustering method, which means that initially every model is in its own cluster. The dissimilarity *d*(*C*_1_,*C*_2_) between two clusters *C*_1_ and *C*_2_ is calculated as the minimal symmetric error $\min _{x \in C_{1}, y \in C_{2}} E_{T}(x, y)$. The two clusters with the lowest dissimilarity are combined into one cluster at a hight given by their dissimilarity. Clusters are iteratively combined until only one cluster remains. This stepwise process can be visualized in a dendrogram [20]. A dendrogram provides a complete description of the single linkage clustering. From such dendrograms it is apparent which models are most similar and which models are more different.

We then color the dendrogram according to the used reduction. Each reduction is mapped to a color and each leaf of the dendrogram receives the color associated to its reduction. Model reductions that cluster together with the original model do not change the model behaviour, while model reductions that are separated from the original model changed the model behaviour. So if the dendrogram separates colors, we consider the model reduction that causes the separation to change the model behaviour. The reduced models that are distributed in a similar way as the original model in the dendrogram are considered to be consistent for the given parameter uncertainty.

In order to analytically compare the distributions of different models in the dendrogram, we calculate the positions in the dendrogram for each model. We then use the test statistics of a Kolmogorov-Smirnov test [21] between a given model and the full model as score for the model. For a given threshold *α*, we say that models with a score lower than the threshold are consistent with the full model at threshold *α*. Finally, the best reduced model is then chosen to be the consistent model that uses the most reductions. In the case of several consistent models having the same number of reductions, the best model is the one with the lowest score. For the remainder of this article we use a threshold of *α*=0.2.

### Simple example

**Fig. 1:**
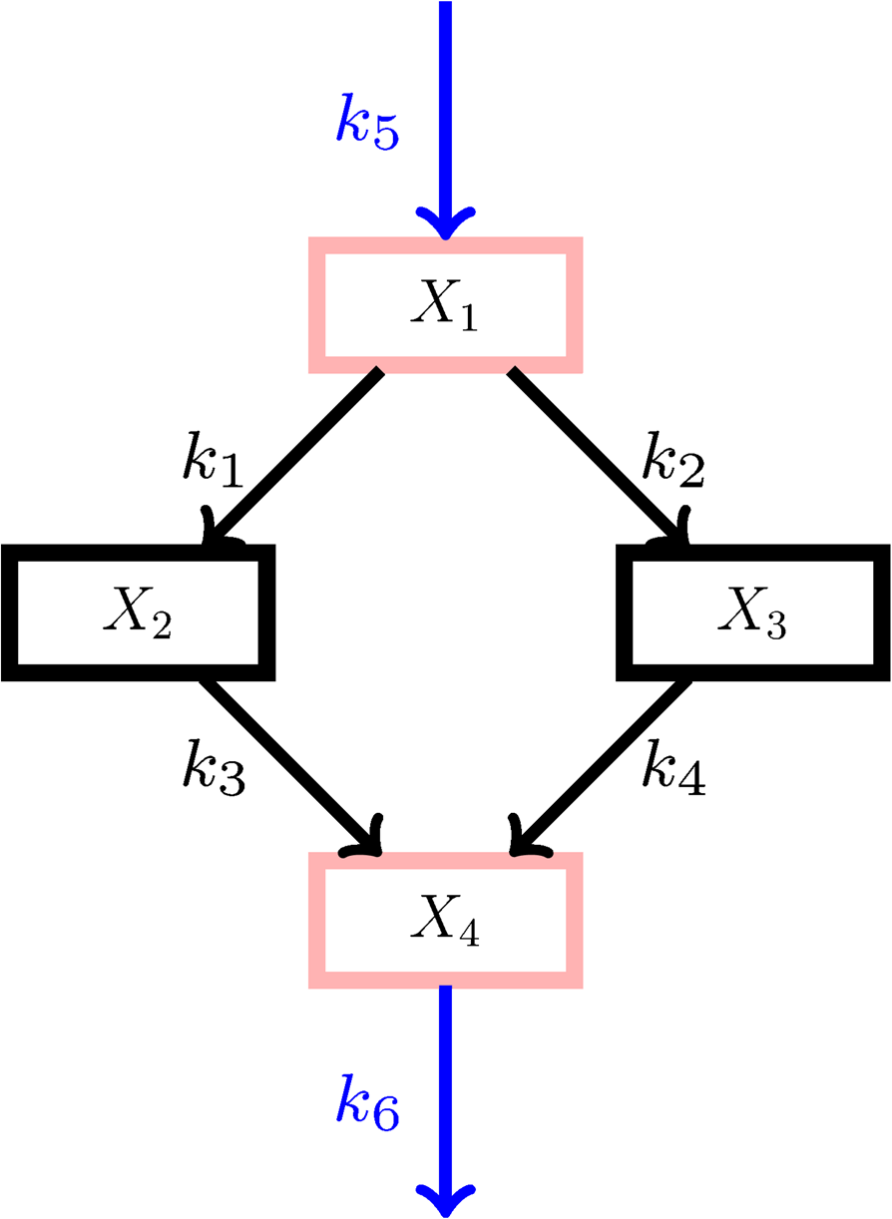
Example network. Each node is a compound and each arrow a reaction. The *k*_*j*_’s are the kinetic parameters of the reactions. Important compounds $\mathcal {M}_{\mathrm {I}}$ and candidate compounds for reduction are specified by pink and black rectangles, respectively. External fluxes are indicated by blue arrows

To illustrate the method, we created a small example network consisting of four compounds as shown in Fig. 1. Each compound occurs only one place in the network and never in combination with other compounds, implying that the complexes are just the compounds. The set $\mathcal {M}_{\mathrm {I}}$ of important compounds is chosen to be number 1 and 4 such that the intermediate compounds 2 and 3 are considered for reduction.

We apply mass action kinetics. Then *k*_*j*_ is the only kinetic parameter of reaction *j*. In the notation of [14] introduced earlier in the article, we have the matrices

5 $$ Z \!= \!\left[\begin{array}{llll} 1 & 0 & 0 & 0 \\ 0 & 1 & 0 & 0 \\ 0 & 0 & 1 & 0 \\ 0 & 0 & 0 & 1 \end{array}\right] B\! =\! \left[ \begin{array}{rrrr} -1 & -1 & 0 & 0 \\ 1 & 0 & -1 & 0 \\ 0 & 1 & 0 & -1 \\ 0 & 0 & 1 & 1 \end{array}\right] Z_{\mathcal{S}} =\left[\begin{array}{llll} 1 & 1 & 0 & 0 \\ 0 & 0 & 1 & 0 \\ 0 & 0 & 0 & 1 \\ 0 & 0 & 0 & 0 \end{array}\right]  $$

for the network. Using mass action we have *d*_*j*_(**x**)=1 such that (2) becomes

6 $$ v_{j}(\mathbf{x})=k_{j}\exp\left(Z_{\mathcal{S}_{j}}^{T}\text{Ln}(\mathbf{x})\right), j\in\left\lbrace1,2,3,4\right\rbrace  $$

for the internal fluxes of **v**. The boundary fluxes are given by

7 $$ \mathbf{v}_{b}= \left[ \begin{array}{llll} k_{5} & 0 & 0 & -k_{6}x_{4} \end{array}\right]^{T}  $$

where the last entry is negative since the flux is leaving the network.

The dynamics are now given by (1) and we have six kinetic parameters *k*_*j*_ associated with one of the six fluxes each. We sampled several parameter sets, which as expected lead to different reduction results. The parameter set that was chosen as reference because it gives particularly interesting reduction results is shown in Table 1. Then, 100 new parameter sets were sampled using this reference set by assuming the parameters to be independently log-normally distributed with the logarithm of the reference values as mean on the log scale and 0.1 as log standard deviation. We applied the reference initial values for all of the parameter sets, and the models were then reduced and clustered as described above.

**Table 1:** Initial values and reference kinetic parameter values for the example network of Fig. 1

Parameter	Value	Initial value	Value
*k*_1_	0.44	*x*_1_(0)	0.4
*k*_2_	0.03	*x*_2_(0)	0.0
*k*_3_	0.55	*x*_3_(0)	0.5
*k*_4_	0.44	*x*_4_(0)	0.4
*k*_5_	0.42		
*k*_6_	0.33		

### Yeast glycolysis example

**Fig. 2:**
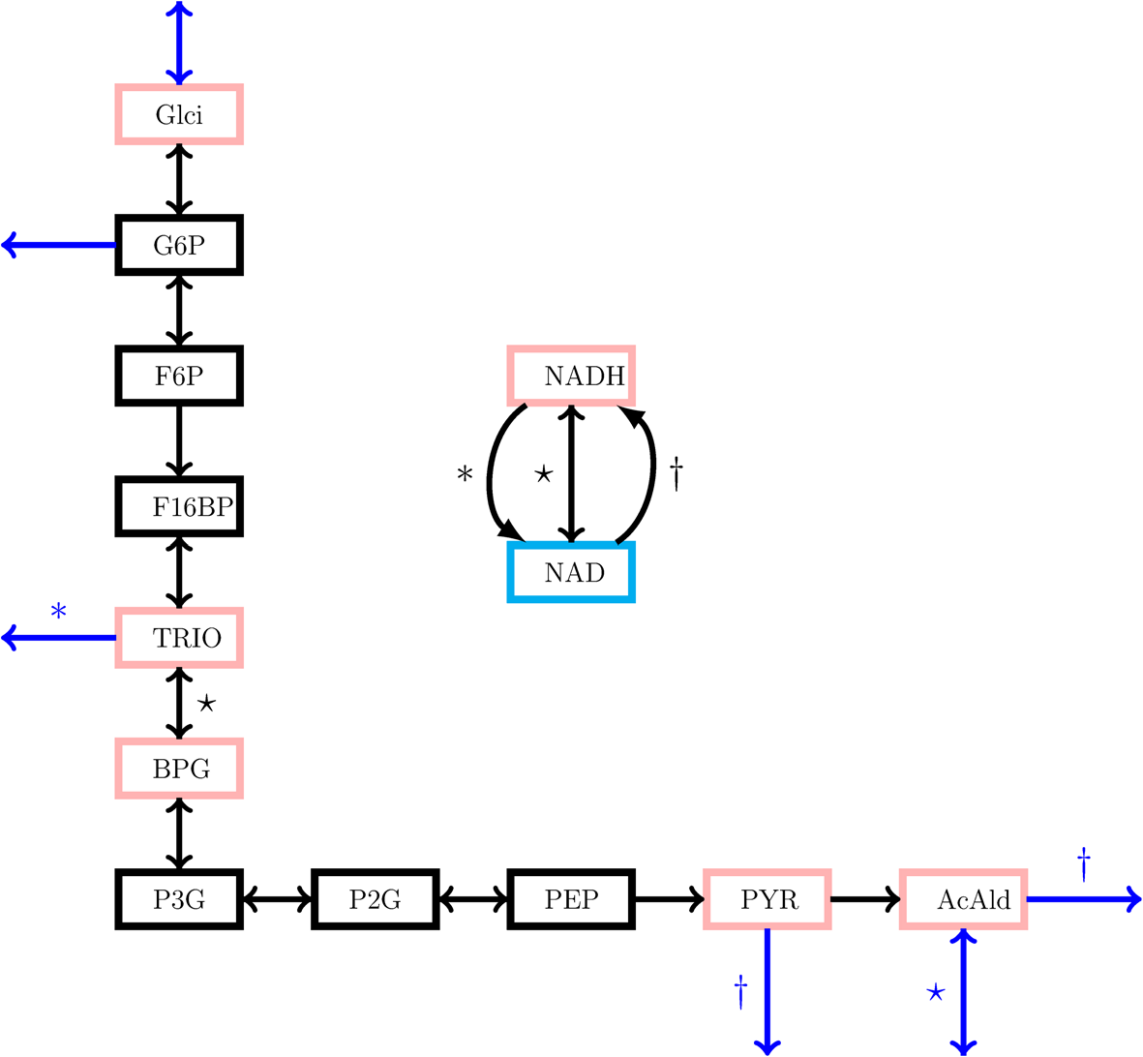
Yeast glycolysis network. Each node is a compound and each arrow a reaction. Important compounds $\mathcal {M}_{\mathrm {I}}$ and candidate compounds for reduction are specified by pink and black rectangles, respectively. NAD indicated by a cyan rectangle is not explicitly included in the model as the total amount of NAD and NADH is conserved. External fluxes are indicated by blue arrows. ∗, ⋆ and *†* show an irreversible reaction from NADH to NAD, a reversible reaction between NADH and NAD and an irreversible reaction from NAD to NADH, respectively. As indicated in the network, different types of these reactions bind to some of the fluxes

We also tested our method on a kinetic model of yeast glycolysis [22] shown in Fig. 2. This model was used in Rao et al. [14] to demonstrate the model reduction method which ignores parameter uncertainty. The model is asymptotically stable around the steady state and the governing equations of the system can be represented in the form of Eqs. 1 and (2) such that the reduction procedure can be applied. The important compounds to form $\mathcal {M}_{\mathrm {I}}$ are Glci, TRIO, BPG, PYR, AcAld and NADH. Accordingly, the six candidates for reduction are F6P, G6P, P2G, P3G, PEP and F16BP, which leads to a total of 2^6^=64 possible reductions for a given parameter set including the full model.

The model has 89 parameters for the different reactions of the network. Each of these parameters should be non-negative, and have a reference value used in [14]. To study the effect of parameter uncertainty on the reduction we sampled parameter sets using these reference values. We assumed the parameters to be independently log-normally distributed with mean equal to the reference value and standard deviation equal to the reference value divided by a scaling parameter. The parameters with reference value zero were set to zero in the sampling. We sampled 100 parameter sets for each of the values 3, 5, 10, 20, 50 and 100 of the scaling parameter. For each of the parameter sets we performed model reduction and clustered all the 100·64=6,400 resulting models for each scaling parameter as described above. We ended up with six dendrograms containing 6400 models each.

In order to check the sensitivity of the method to the number of parameter sets sampled, we also sampled 1000 parameter sets for the model with scaling parameter 50. For each parameter set we considered all model reductions with a Kolmogorov-Smirnov test score below a threshold of 0.5 for the 100 previous parameter sets. We performed model reduction and clustering as above.

All analyses were performed in MATLAB [23]. All code used to generate the results is available in the online supplementary material.

## Results

### Simple example

**Fig. 3:**
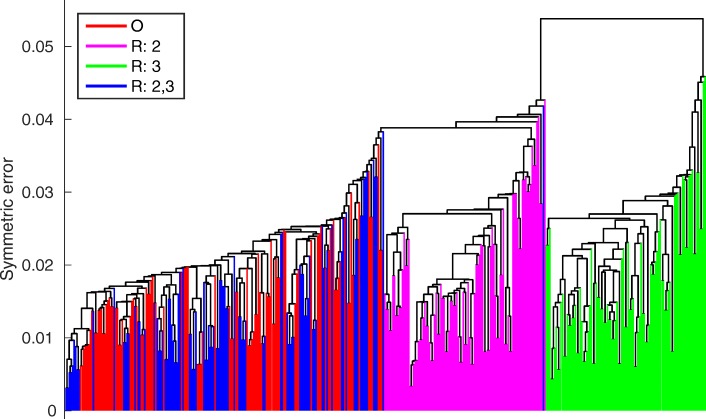
Model clustering. Dendrogram from single linkage clustering of all model reductions with 100 parameters sets. Parameters were sampled from a log-normal distribution with log standard deviation 0.1

For the used parameter values, the model with both compounds number 2 and 3 reduced clustered together with the original model and had a Kolmogorov-Smirnov score of 0.17. Both the model with only compound 2 removed and the model with only compound 3 removed had a Kolmogorov-Smirnov score of 1.00. The models with only compound 3 reduced were the furthest from the cluster including the original model. Figure 3 shows the single linkage cluster dendrogram. The behaviour changes substantially for different parameter values and parameter uncertainties.

### Yeast glycolysis example

**Fig. 4:**
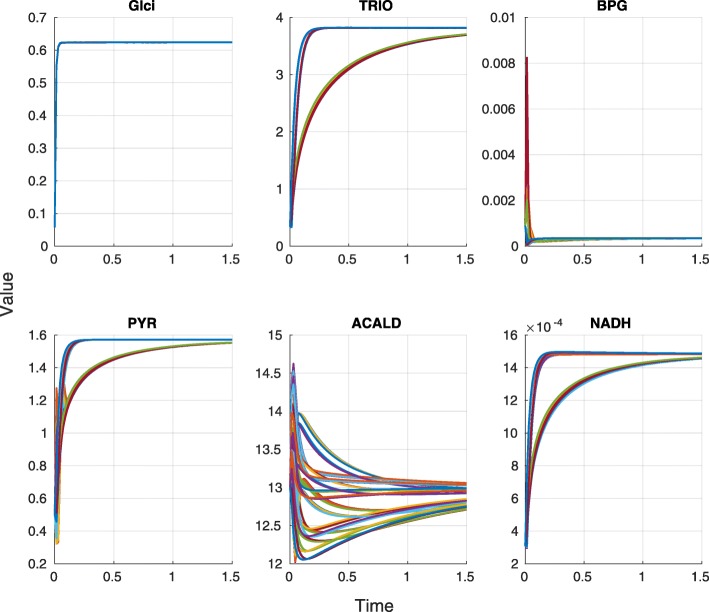
Yeast trajectories. The $\mathcal {M}_{\mathrm {I}}$ states are shown for the reference parameters. Each color corresponds to a different model reduction

The trajectories of the full model and all reduced models using the parameter set from [14] show no effect for Glci, two groups for TRIO, PYR and NADH, but no clear picture for BPG and ACALD (Fig. 4). For the reference parameter set, we found two big clusters. The first cluster contained the full models as well as all the models with compound F16BP not reduced, and the second cluster contained all models with F16BP reduced.

**Fig. 5:**
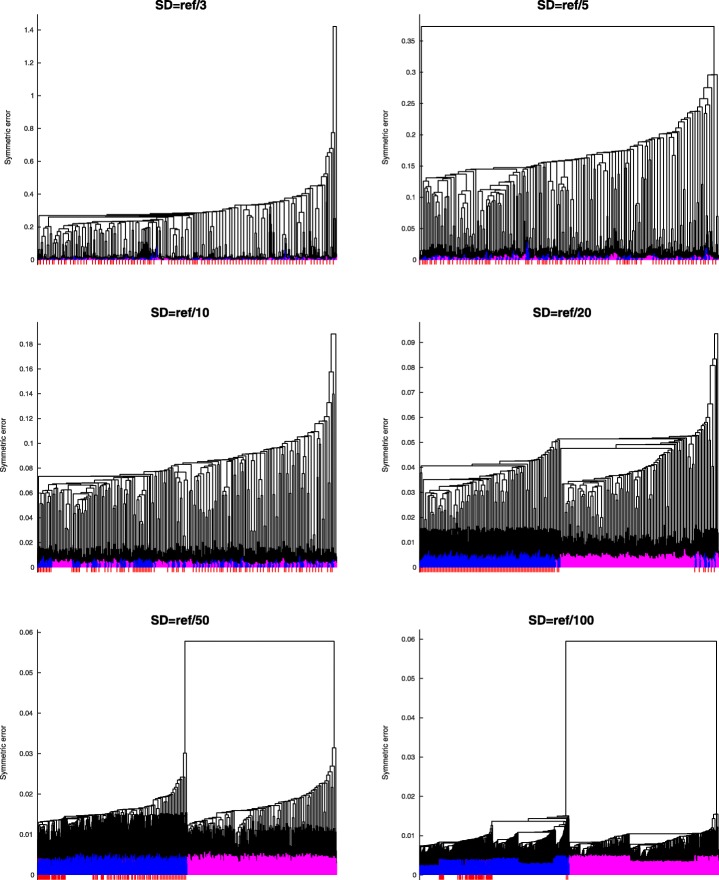
Model clustering of F16BP reduced models. Dendrogram from single linkage clustering of all the model reductions using 100 parameters sets. Parameters were sampled from a log-normal distribution with standard deviation as reference value divided by 3 (top left), 5 (top right), 10 (center left), 20 (center right), 50 (bottom left) and 100 (bottom right). The original models are shown in red, models where F16BP was reduced are purple and all other models are blue

The clusterings for a distribution of parameters depended on the parameter distribution. When the standard deviation was high, there were no clear clusters and the full models were evenly distributed between the reduced models (Fig. 5, top left). This means that the uncertainty in the parameters had more effect than the model uncertainty due to reduction. The more certain the parameters were, the more we saw a clear picture emerge, with all models that had compound F16BP reduced clustering together and all other models forming a separate cluster (Fig. 5, top right, bottom left). When decreasing parameter uncertainty even further, the original models started forming a cluster of models where both compounds PEP and F16BP were not reduced (Fig. 5, bottom right).

**Fig. 6:**
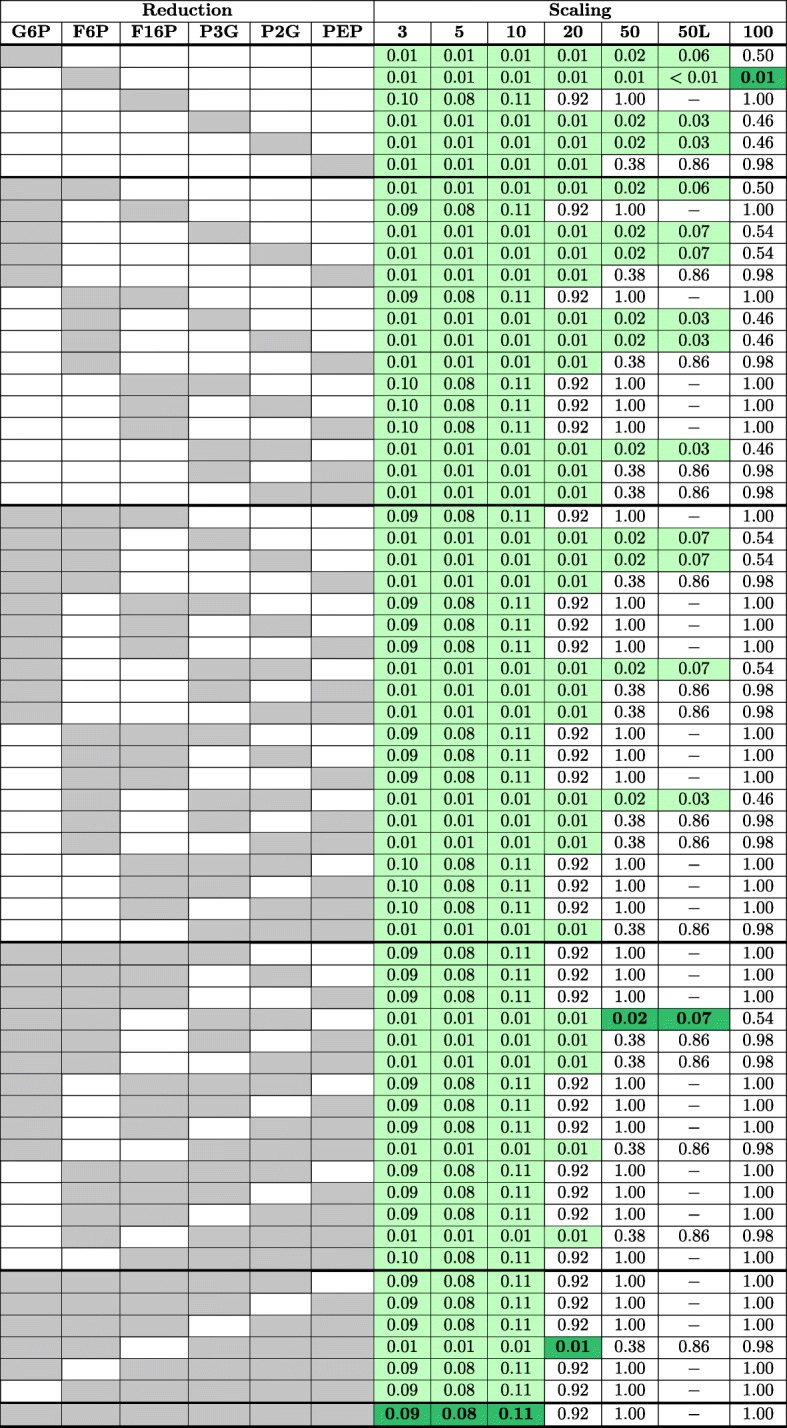
Kolmogorov-Smirnov test scores. Kolmogorov-Smirnov test scores for all the model reductions using 100 parameters sets as well as the sensitivity analysis with 1000 parameter sets (50L). The compounds in gray are reduced in the model of the corresponding row. Parameters were sampled from a log-normal distribution with standard deviation as reference value divided by the scaling factor. Models that are consistent with the original model are shown in light green and the best reduced model for each case is shown in dark green

In addition to finding clusters that are inconsistent with the model uncertainty, we studied the distribution of the reduced models in the dendrogram. In the case of large parameter uncertainty (scaling parameters 3, 5, 10) the distribution of the fully reduced model in the dendrogram was similar to the distribution of the original model (Kolmogorov-Smirnov 0.11 or smaller). In the case of relatively large uncertainty (scaling parameter 20), all the models that did not reduce F16BP were distributed similarly to the original model (Kolmogorov-Smirnov 0.01). When the uncertainty was relatively low (scaling parameter 50), all models with F16BP and PEP not reduced clustered together with the full model (Kolmogorov-Smirnov 0.01 or 0.02). However, in the case of very low uncertainty (scaling parameter 100) the only model whose distribution in the dendrogram was similar to the distribution of the original model was the one where only F6P was reduced (Kolmogorov-Smirnov 0.01). The sensitivity analysis showed that whether or not a reduction was consistent for a given uncertainty did not dependent on the number of parameter sets (Fig. 6).

## Discussion

We developed a new method to evaluate model reductions under parameter uncertainty based on the symmetric error measure in (4). In the yeast glycolysis example we showed that the amount of parameter uncertainty influences the model reduction. In particular, model uncertainty and parameter uncertainty are positively related. When the model parameters are uncertain, the model can be reduced further without increasing uncertainty in the model dynamics. We have also demonstrated empirically that if a model can be reduced to a certain degree for a given amount of uncertainty, then it can be reduced to at least the same degree if the uncertainty increases. If a model is used to analyse different scenarios, the parameters for all the scenarios should be considered when reducing a model. A full model should only be reduced to a model that is consistent for all considered scenarios. In addition to parameter values, uncertainty in initial values should also be considered. Our analysis shows that the reduction of Rao et al. [14] for the yeast model agrees with our best reduction for a relatively high amount of uncertainty, but becomes inappropriate for low or very large uncertainty.

In the simple example we demonstrated that it is sometimes better to reduce two complexes than just one. This also shows that even without parameter uncertainty the iterative approach used in [14] may not find the best reduction. Whether or not the best reduction is found depends on the symmetric error cut-off value. In the example, the reduced model would be found with symmetric error cut-off value at least 0.04, even though the symmetric error is only 0.02. The reference values in Table 1 for the parameters were chosen to illustrate this behaviour.

The novelty of our approach is a new way to evaluate model reduction. This model reduction evaluation criterion can be applied together with any model reduction method. Our criterion does not assume that the full model with a given parameter set is optimal. Instead it compares the full model with a wide range of parameter values to reduced models with the same range of parameter values to find a reduced model with the same properties, including model uncertainty. A reduced model with lower uncertainty in the trajectories could lead to overconfidence in the results.

A limitation of our method is that we need to choose a set $\mathcal {M}_{\mathrm {I}}$ of important compounds. This choice is subjective and affects the resulting reduced model. However, there are some natural choices for the set $\mathcal {M}_{\mathrm {I}}$, which depend on the model purpose. Of course $\mathcal {M}_{\mathrm {I}}$ should contain all the compounds the study is investigating. It should also contain all the compounds whose concentrations are measured experimentally. Another limitation of our approach is that we have to choose the length *T* of the time series. It is important that at time *T* the trajectories are close to the steady state, because otherwise the error integral does not cover the entire model dynamics. On the other hand *T* should not be too large because otherwise the error integral reduces to the difference in steady states. If the model does not approach a steady state the dissimilarity measure we use may not be appropriate. There may also be some scaling issues with our proposed approach. Already in the case where we have to evaluate 64 models, we have to calculate a 6400×6400 matrix of dissimilarity measures using 100 parameter sets. For most practical examples, however, it is possible to reduce the sample space of reductions to a manageable size. In our sensitivity analysis with 1000 parameter sets, we have solved the issue by using the first 100 parameter sets to exclude some model reductions, which lead to a 32,000×32,000 dissimilarity matrix. The calculation of this matrix is the computational bottleneck of the method, but parallel computing can be applied. Moreover, it is possible to iteratively compare only a few models at a time. We suggest that investigators adapt their strategies for model reduction based on model size, complexity and choice of the set $\mathcal {M}_{\mathrm {I}}$. The Kolmogorov-Smirnov score leads to an automatic way of choosing the best reduced model. However, we believe that it is important to look at the dendrograms and not choose the model reduction only based on the Kolmogorov-Smirnov scores.

## Conclusions

We presented a new method for evaluating models under parameter uncertainty and applied it for comparing full models to reduced models. We showed that multiple reductions can result in better models than individual reductions and that the amount of parameter uncertainty influences the choice of reduced models.

## Additional file

Additional file 1 Scripts. Archive file containing all the scripts needed to produce and analyse the data of this paper. These scripts also produce Figs. 3, 4, and 5. (ZIP 23.3 kb)

## Abbreviations

ODE: Ordinary differential equation
